# Osteoclast activity sculpts craniofacial form to permit sensorineural patterning in the zebrafish skull

**DOI:** 10.3389/fendo.2022.969481

**Published:** 2022-11-01

**Authors:** Kelly Z. Miao, Austin Cozzone, Joana Caetano-Lopes, Matthew P. Harris, Shannon Fisher

**Affiliations:** ^1^ Department of Pharmacology and Experimental Therapeutics, Boston University Aram V. Chobanian & Edward Avedisian School of Medicine, Boston, MA, United States; ^2^ Department of Orthopaedic Surgery, Boston Children’s Hospital, Boston, MA, United States; ^3^ Department of Genetics, Harvard Medical School, Boston, MA, United States

**Keywords:** osteoclast, neuromast, lateral line, foramen, live imaging, zebrafish, craniofacial development

## Abstract

Efforts to understand the morphogenesis of complex craniofacial structures have largely focused on the role of chondrocytes and osteoblasts. Along with these bone–creating cells, bone–resorbing osteoclasts are critical in homeostasis of adult skeletal structures, but there is currently limited information on their role in the complex morphogenetic events of craniofacial development. Fundamental aspects of skull formation and general skeletal development are conserved from zebrafish to mammals. Using a *cathepsinK* reporter, we documented osteoclast location in the developing zebrafish skull over several weeks, from 5.18 mm to 9.6 mm standard length (approximately 15 to 34 days post fertilization). While broad distribution of osteoclasts is consistent across individuals, they are sparse and the exact locations vary among fish and across developmental time points. Interestingly, we observed osteoclasts concentrating at areas associated with neuromasts and their associated nerves, in particular the hyomandibular foramina and around the supraorbital lateral line. These are areas of active remodeling. In contrast, other areas of rapid bone growth, such as the osteogenic fronts of the frontal and parietal bones, show no particular concentration of osteoclasts, suggesting that they play a special role in shaping bone near neuromasts and nerves. In *csf1ra* mutants lacking functional osteoclasts, the morphology of the cranial bone was disrupted in both areas. The hyomandibular foramen is present in the initial cartilage template, but after the initiation of ossification, the diameter of the canal is significantly smaller in the absence of osteoclasts. The diameter of the supraorbital lateral line canals was also reduced in the mutants, as was the number of pores associated with neuromasts, which allow for the passage of associated nerves through the bone. Our findings define important and previously unappreciated roles for osteoclast activity in shaping craniofacial skeletal structures with a particular role in bone modeling around peripheral cranial nerves, providing a scaffold for wiring the sensioneural system during craniofacial development. This has important implications for the formation of the evolutionarily diverse lateral line system, as well understanding the mechanism of neurologic sequelae of congenital osteoclast dysfunction in human craniofacial development.

## Introduction

Craniofacial skeletal structures must create strong protection for the brain while allowing the peripheral nervous system to relay sensory inputs from the environment (1, 2). Disruptions to these processes underlie craniofacial birth defects, which broadly encompass the most common structural congenital defects in humans (3–5). In the context of understanding skeletal development of the skull, much of current research focuses on the evolutionary origin, growth and function of cells which build skeletal structures, such as chondrocytes and osteoblasts (6, 7) and in describing structural form and shape of individual bones during development and how they functionally integrate (8, 9). However, the form of individual bones and their final structural role also relies on the activity of bone-resorbing osteoclasts during development (10, 11). Relatively little is known of how these cells shape craniofacial development.

Osteoclasts are a specialized cell type in the hematopoietic lineage and can persist as mononucleate cells or form multinucleated cells by fusion of precursor cells (12, 13). Osteoclasts resorb bone in a tightly regulated homeostatic relationship with bone–generating osteoblasts to maintain general morphology and internal structure of bones in adulthood (14). Osteoclasts are typically stimulated by RANK/RANKL (15) and inhibited by OPG (16). Significant deviations from this homeostatic relationship contribute to adult diseases such as osteoporosis, arthritis, Paget’s disease and periodontitis (17–19). Teleosts such as medaka have been successfully used to model the osteoclast dysfunction seen in these disorders (20, 21). Mutations leading to osteoclast dysregulation or dysfunction are the major cause of juvenile osteopetrosis (22–24), which can include significant neurologic sequelae. Understanding facial canal dehiscence caused by developmental abnormalities is important due to consequences for patients such as facial paralysis and hearing loss (25, 26), similar issues have been observed in relation to nerve compression by irregularities in the supraorbital foramen resulting in severe headaches (27, 28). There have been prior descriptions of the normal developmental role of osteoclasts in specific locations in the skull suggesting their overall importance (29–33).

The brain and associated cranial nerves are established prior to the formation of mineralized bone (9, 34, 35, 36) and need to be appropriately scaffolded to allow for function. This interaction adds further complexity to modeling craniofacial form. There is strong evidence in humans and other mammals that appropriate bone development and morphogenesis requires crosstalk between nerves and bone tissue (37–41). Osteoclasts possess nicotinic acetylcholine receptors (42) with agonists of these receptors causing apoptosis of osteoclasts (43) and the β2-adrenergic receptor has been shown to indirectly impact osteoclasts by modulating RANKL (44). Zebrafish provides an efficient means to model these interactions (45–47). The influence of nerves on the craniofacial skeleton has been established in both zebrafish and humans (39, 48). The zebrafish skull houses both cranial nerves and the anterior lateral line (aLL). The lateral line is a sensory system of aquatic vertebrates consisting of neuromasts with mechanosensory hair cells that sense changes in water pressure (49). In zebrafish, the afferent nerves of the cranial neuromasts pass through the hyomandibular foramen, which also houses the facial and auditory cranial nerves (50). After metamorphosis, as the fish transition into adulthood, a subset of cranial neuromasts and nerves become encased in bony lateral line canals (51).

To describe the normal distribution of osteoclasts during craniofacial development, we used a *cathepsinK* transgenic line to carry out serial confocal microscopy on developing zebrafish to localize osteoclasts in craniofacial structures. We find that osteoclasts during development are not distributed evenly and are absent from some highly dynamic structures, including the osteogenic fronts of the frontal and parietal bones. Instead, they cluster densely around areas associated with cranial nerves and sensory cells, including the aLL and the facial nerve (cranial nerve VII). Furthermore, mutants lacking osteoclasts (52) have dysregulation of neurological access and scaffolding, suggesting that bone remodeling, involving high levels of osteoclast activity, is important for specific functional attributes of cranial morphology. Our results highlight the important role of nervous system–bone crosstalk and show that the zebrafish provides a viable model system to understand these interactions.

## Materials and methods

### Animal husbandry and care

All zebrafish (*Danio rerio*) used for this study were maintained according to standard protocols (53). All experiments were conducted in strict accordance with the Guide for the Care and Use of Laboratory Animals of the National Institutes of Health and all protocols were approved by the Institutional Animal Care and Use Committee at Boston University. The transgenic line *Tg(Ola.ctsk:FRT-DsRed-STOP-FRT,Cre, cmlc2:GFP)* henceforth referred to as *ctsk:dsRed* consists of a medaka promoter active in osteoclasts (54) combined with a *cmlc2* heart marker for early embryo screening. The generation of the *ctsk:dsRed* and *csf1ra^mh5^* mutant have been described previously (52).

### Live calcein staining

A stock calcein solution of 2% in dH2O was adjusted to pH 7 (Sigma-Aldrich, cat: C0875) using NaOH. Stock solution was diluted 1:10 in zebrafish system water to generate fresh 0.2% staining solution. As previously described (55), fish were immersed in staining solution for 30 minutes, then transferred to clean water for 5 minutes three times to remove excess calcein. Post staining, fish were directly imaged or placed back onto the system for serial imaging. For successive imaging, fish were restained after each imaging session to integrate dye into newly formed bone.

### Staging and measurements of standard length

Prior to confocal imaging, fish were anesthetized in Tricaine (MS-222, Sigma-Aldrich) and measured as previously described (9). Standard length was used as a proxy for developmental stage (56).

### *In-vivo* imaging

For confocal imaging, fish were mounted in glass bottom dishes (MatTex Corporation) in 2% low melt SeaPlaque agarose in dH_2_O (Lonza Catalog #: 50115). Once the agarose solidified within the dishes the fish were covered in fish water. The areas around the gills and mouth were carefully cleared of agarose with a dissecting probe to allow respiration. Total imaging time was around 10 min, after which fish were removed from the agarose and allowed to recover in water. Daily imaging of identified individual fish was conducted during early phases of rapid bone growth, and later reduced to once every 2-3 days as growth slowed (9). Individuals were imaged using the Leica TCS-LSI III macro-confocal microscope with 2× and 5× Plan APO objectives, generating.lif files which were converted into.tiff files using Fiji/ImageJ for further analysis. A total of 84 individual z-stacks were generated over a period of 22 days. Serial live-imaging is generally well tolerated as described previously (9). In this series one out of six original individuals failed to survive through the imaging period and required replacement with a sibling.

### Alizarin red staining, dissection and imaging of fixed bone

Fish were fixed overnight in 4% PFA in PBS and Alizarin red staining was performed as previously described in the literature (57). Samples were then dissected to remove the bones from remaining tissue, mounted in 2% low melt agarose then imaged on the Leica TCS-LSI III macro-confocal microscope as described above.

### Osteoclast overlay image creation

For composites, the imaging protocol was followed except that groups of individuals were matched by standard length and imaged at only one stage. To generate the overlaid images, max projections of the *ctsk:dsRed* channel were generated from the size-matched imaging files. These were processed in Fiji to despeckle, then threshold adjusted to obtain representations of osteoclast area, shape and location. Each individual was assigned a distinct color; the colorized images were overlaid in Adobe Photoshop on a greyscale max projection of the calcein–stained bone from a representative fish.

### Quantification of bone pores

Maximum intensity projections of the calcein or Alizarin red staining were generated in Fiji. and adjusted for brightness and contrast. The pore diameters were obtained through manual tracing and measured in Fiji; statistical analysis was carried out in GraphPad Prism 9.

### 3D volume rendering of canals

Z-stacks were loaded into Fiji and adjusted for brightness/contrast. Canal limits were manually traced on selected images in the stack using the Segmentation Editor plugin, the interpolation function was then used to predict canal areas on images in between the manual traces in the Z-stack. 3D renderings were then created using the 3D Viewer function and images/videos were exported. For both still images and videos of the 3D rendering angles of imaging between both WT and mutants were matched.

## Results

### Imaging of *ctsk:dsRed* transgenic fish shows variations in osteoclast distribution in the developing skull

**Figure 1:**
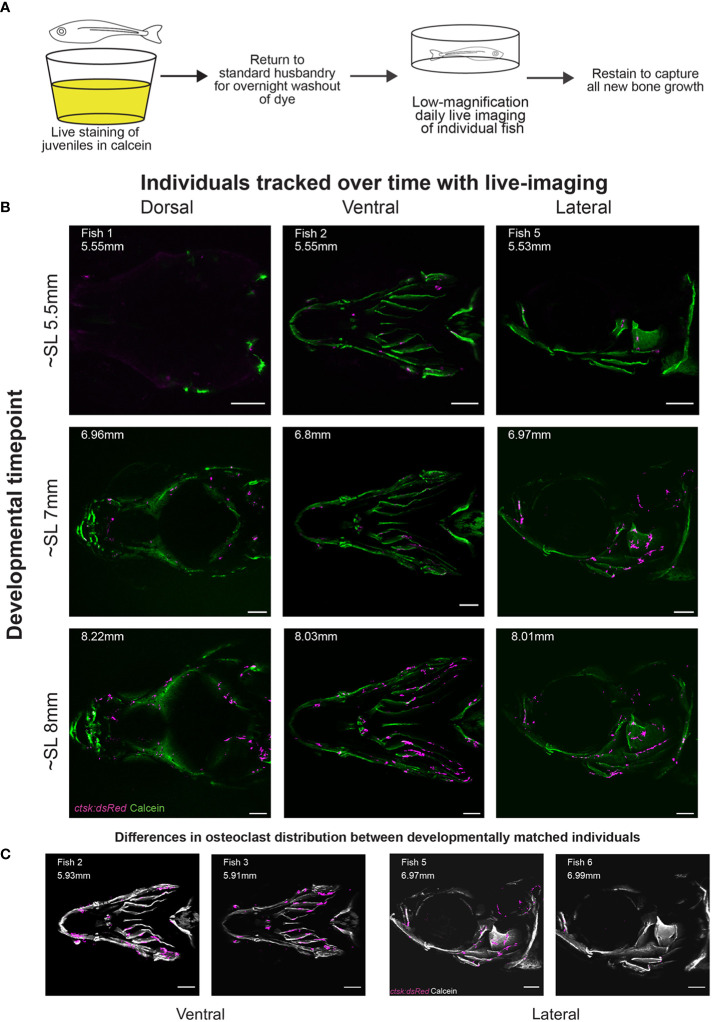
Daily live imaging shows changes in osteoclast distribution. Osteoclasts in six individuals were tracked throughout initiation and early growth of bone in the skull, all scale bars represent 200μm. **(A)** Diagram representing the workflow for daily live imaging of the transgenic *ctsk:dsRed* fish, fish were individually housed and then stained with calcein to capture bone growth, then returned to husbandry system to allow for both washout of dye and regular feedings between each daily live imaging session. **(B)** Osteoclast location and distribution is shown on calcein stained mineralized bone. Differences can be tracked within individuals over time and imaging capturing the dorsal, ventral and lateral angles allows for the tracking of osteoclasts on the entirety of the skull **(C)** Representative osteoclast distributions in magenta are displayed on a greyscale image of calcein staining showing that when comparing between individuals, though matched by developmental timepoint (as shown in SL), there is variation in distribution.

Previous studies of osteoclasts during development have generally captured information on cell distribution at single timepoints in fixed and stained individual animals. Using a previously described low-magnification confocal imaging methodology (9), we visualized the localization of osteoclasts during skull development in individual zebrafish over time. We used *ctsk:dsRed*, a previously established transgenic line which marks functional osteoclasts (52) combined with repeated calcein staining to capture bone growth throughout development (**Figure 1A**).

We imaged osteoclasts in six fish through live serial confocal microscopy, with individuals assigned into groups covering the ventral (3 individuals, 1 replacing an early death), dorsal (1 individual) or lateral (2 individuals) angles. Fish were imaged from 15dpf to 34dpf, initially on a daily basis, then transitioning to once every 2-3 days as growth slowed at later stages. This allowed us to follow the development of individuals ranging from SL5.18mm to SL9.6mm, generating 84 individual z-stacks with 17 z-stacks taken per individual; in the case of the early death, 10 initial z-stacks were completed and then a sibling replacement was imaged for an additional 6 z-stacks. The original imaging files are available for public access in the FaceBase database (**DOI:** 10.25550/1-X62C). Comparison of osteoclast distribution within individuals at different timepoints and between individuals (**Figure 1B**) shows that osteoclasts are sparse relative to the cells that make up the underlying bone. This sparsity is notable at the osteogenic fronts of the frontal and parietal bones, previously established as areas of high osteoblast activity (58). This implies that *ctsk+* osteoclasts do not play a significant role in shaping the morphology of those regions of active bone growth. Comparisons of osteoclasts in different individuals imaged from the same angle at matched developmental timepoints (**Figure 1C**) also show variability in osteoclast distribution between developmentally matched individuals. This stands in contrast with osteoblasts which have a highly regular pattern between individuals throughout development (9).

### Osteoclasts aggregate in specific areas during development across individuals and are associated with nerves and sensory cells

**Figure 2:**
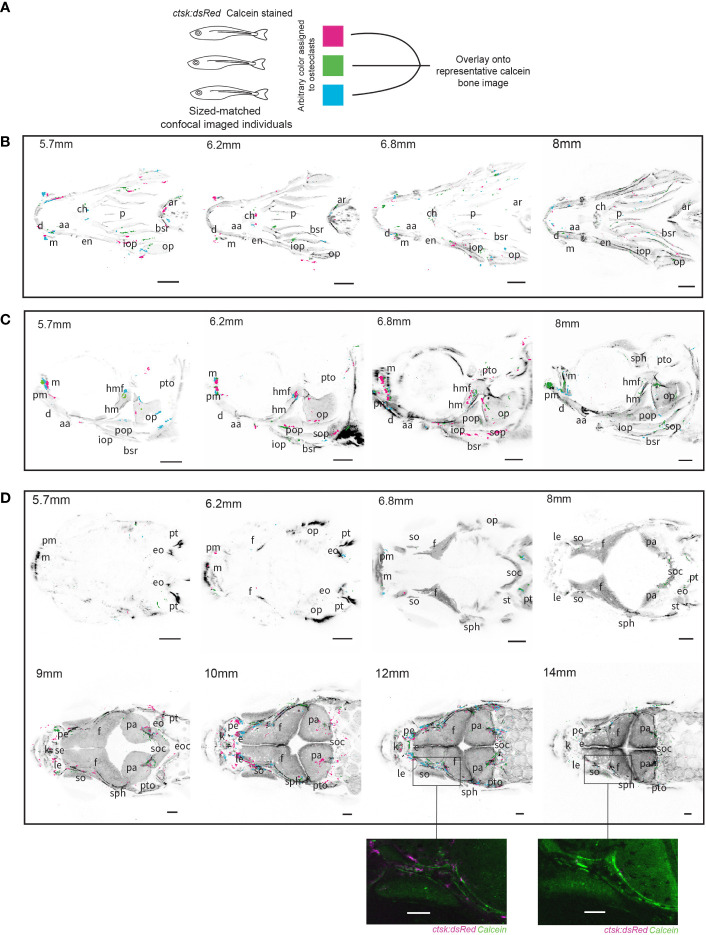
Overlays of osteoclasts from imaged individuals shows patterns in distribution. Three individuals were size-matched, stained and live-imaged to create each representation, all scale bars represent 200μm. **(A)** Diagram representing the methodology used to combine the three individuals in each image. **(B)** Ventral imaging shows that osteoclasts are distributed along the branchiostegal rays (bsr) throughout all of the tracked developmental timepoints, in addition concentrations of osteoclast can be seen on the mandibles (m) which house lateral line neuromasts **(C)** Lateral imaging of osteoclasts shows the same distributions as seen on the branchiostegal rays and mandibles but 6.8mm and 8mm images show concentrations of osteoclasts specifically around the hyomandibular foramina (hmf) and on the opercle (op) **(D)** Dorsal imaging of osteoclasts shows that osteoclasts are not highly active prior to growth of the frontal (f) and parietal bones (pa), with a notable concentration of osteoclasts around the supraorbital lateral line canals which run through the frontal bones around 10mm and 12mm which is comparatively reduced by 14mm, a detailed comparison of individuals at 12mm and 14mm is shown in unprocessed form. Abbreviations: aa (anguloarticular), ar (arches), bsr (branchiostegal ray), ch (ceratohyal), d (dentary), en (entopterygoid), eo (epioccipital), e (ethmoid), eoc (exoccipital), ff (facial nerve foramen), f (frontal), hm (hyomandibula), iop (interopercle), k (kinethmoid), le (lateral ethmoid), m (maxilla), op (opercle), p (parasphenoid), pa (parietal), pe (preethmoid), pm (premaxilla), pop (preopercle), pt (posttemporal), pto (pterotic), sph (sphenotic), se (supraethmoid), soc (supraocciptal), so (supraocciptal), so (supraorbital), sop (subopercle), st (supratemporal).

Through the course of the serial live imaging, we observed general patterns of osteoclast distribution across multiple individuals. To better visualize these patterns, we imaged groups of size–matched individuals and then overlaid osteoclast distributions (**Figure 2A**). The original imaging files used to create the composites are available for public access in the FaceBase database (**DOI:** 10.25550/6-F6XM). A table with nomenclature of bone structures in the skull is displayed in **Table 1**. Osteoclast clustering was first apparent at SL5.7mm along the branchiostegal rays and the mandibles, some of the first bones to mineralize within the zebrafish skull (8). The osteoclasts at these locations were most prominent at earlier stages and diminished during the imaging period (**Figure 2B**).

**Table 1:** Abbreviations for bone structures within the zebrafish skull as seen in **Figure 2** utilizing terminology from Cubbage and Mabee (8).

List of abbreviations for skeletal elements									
**aa**	anguloarticular	**eo**	epioccipital	**iop**	interopercle	**pa**	parietal	**sph**	sphenotic
**ar**	arches	**e**	ethmoid	**k**	kinethmoid	**pe**	preethmoid	**se**	supraethmoid
**bsr**	branchiostegal ray	**eoc**	exoccipital	**le**	lateral ethmoid	**pm**	premaxilla	**soc**	supraoccipital
**ch**	ceratohyal	**ff**	facial nerve foramen	**m**	maxilla	**pop**	preopercle	**so**	supraorbital
**d**	dentary	**f**	frontal	**op**	opercle	**pt**	posttemporal	**sop**	subopercle
**en**	entopterygoid	**hm**	hyomandibula	**p**	parasphenoid	**pto**	pterotic	**st**	supratempora

Companion diagram in **Supplemental Material** indicating location of abbreviated structures showing dorsal (A) at around SL9mm, ventral (B) at around SL8mm and lateral (C) at around 8mm.

Osteoclasts also consistently clustered around the hyomandibular foramen which allows for the passage of the facial and auditory cranial nerves and aLL nerve (50) (**Figure 2C**). This dense concentration of osteoclasts is especially conspicuous around SL6.2mm and SL6.8mm, corresponding to previously described key points in the ossification of this structure from a cartilaginous template (8). Osteoclast activity around the hyomandibular foramen continues at SL8mm after this initial period of mineralization. The timing of osteoclast activity and timing of mineralization suggests that osteoclasts are involved in shaping the morphology of the canals both during and after the transition from cartilage to mineralized bone.

In the dorsal overlays at SL5.7mm and SL6.2mm there were no osteoclasts, consistent with the lack of mineralized bone. There is also little osteoclast activity at the timepoints most closely associated with early rapid growth at the osteogenic fronts of the major bones of the cranium the frontal and parietal bones, between SL6.8mm and SL8mm (9). Starting at SL9mm and continuing into SL10mm, stages when the frontal bones reach their final stages of growth and meet to form sutures, there is a notable increase in osteoclasts around the supraorbital lateral line canals. These canals have been previously observed to start forming with the neuromasts on the epithelium above the dermal bone, these neuromasts then sink down into a depression in the bone which is progressively enclosed by walls of ossifying bone until a closed channel is formed (51, 59). The concentration of osteoclasts continues along these canals at SL12mm but is nearly gone by SL14mm (**Figure 2D**). Our data implies that heightened osteoclast activity and bone resorption is associated with previously described key stages of supraorbital lateral line canal development.

### *csf1ra^mh5^* mutants display changes in the morphology of the hyomandibular foramen

We document a clear spatial and temporal relationship between osteoclasts and the formation and sculpting of structures associated with nerves and sensory cells. We infer that osteoclasts have an important role in the formation of these structures. To support our hypothesis, we examined mutants in *colony-stimulating factor 1 receptor*, a (*csf1ra^mh5^*), a key regulator of the proliferation, differentiation and function of myelomonocytic cells, including osteoclasts (52). As previously described, *csf1ra* mutants lack osteoclasts and other myelomonocytic cells (56, 60).

**Figure 3:**
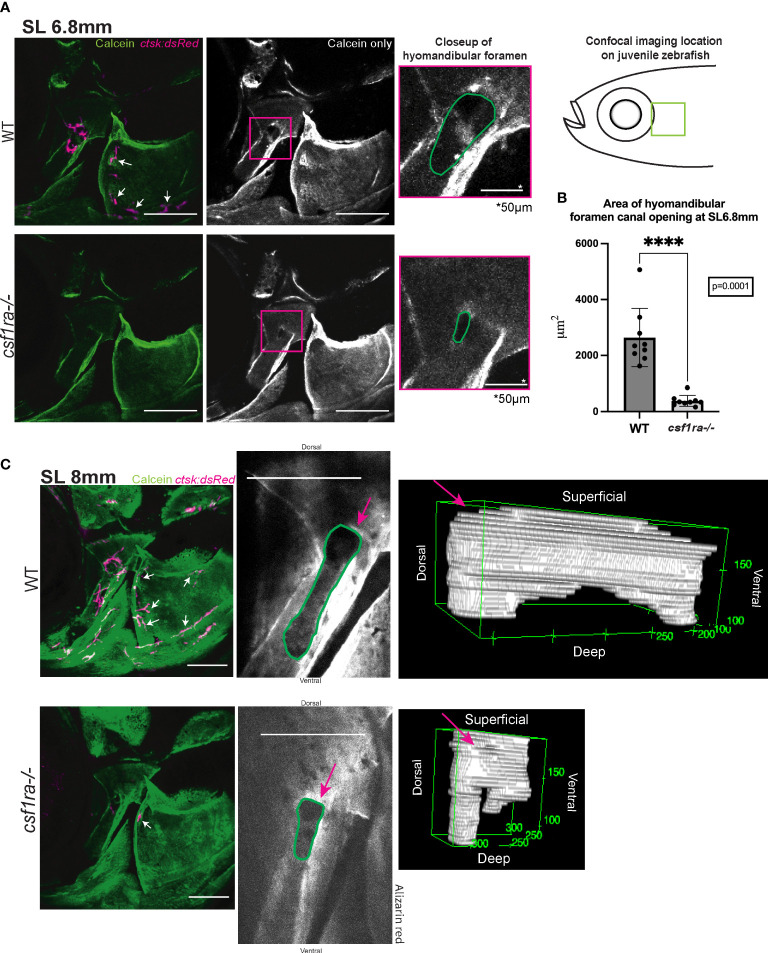
Imaging of hyomandibular foramen using calcein-stained *csf1ra* mutants with *ctsk:dsRed* and their wild-type siblings shows differences in canal opening morphology. Size matched individuals were imaged at SL6.8mm and SL8mm, all images are max projections of collected z-stacks, scale bars represent 200μm unless otherwise indicated. **(A)** Diagram represents location of the confocal imaging conducted on the lateral side of the fish focusing in on area of high osteoclast activity as seen in **Figure 2**. Lack of osteoclast activity in the foramen as seen in *csf1ra* mutants results in morphological differences in the foramen canal opening **(B)** Quantification of area of canal opening (WT n=9, *csf1ra^-/-^* n=9) lines indicate mean with SEM, Mann-Whitney U t-test (two-tailed) used for statistical analysis, 95% confidence interval **** indicates p-value = 0.0001 **(C)** Individuals at SL8mm display differences in canal morphology shown using representative 3D renderings of internal volume inside of the canals of WT siblings and *csf1ra* mutants, videos of these 3D renderings can be found in the **Supplemental Figures**.

Homozygous *csf1ra^mh5^* mutants and wild-type siblings expressing *ctsk:dsRed* were vitally stained with calcein and observed during the period of osteoclast concentration at the hyomandibular foramen. At SL6.8mm the wild-type fish had a larger, more open canal at the hyomandibular foramen (**Figure 3A**), while the canal in the mutants had a significantly smaller cross sectional area (**Figure 3B**). By SL8mm, the canal in the wild–type fish was longer, defined by two deeper areas on each end with a high concentration of osteoclasts. In contrast, the mutants retained a rounder canal opening with a lower overall internal volume and different spacing of the sunken areas. On the surrounding opercle surface, these images also capture small clusters of osteoclasts in the WT fish associated with small additional foramina lacking in the mutants (61) (**Figure 3C**). We also documented close associations between osteoclasts and bone-forming osteoblasts in the hyomandibular foramen at this developmental timepoint (**Supplemental Figure 1**) (61). Our analyses indicate that the osteoclasts serve an essential, and specific role in shaping normal foramen morphology in the hyomandibula and opercle bones.

### *csf1ra^mh5^* mutants lack pores associated with the neuromasts of the supraorbital lateral line canals

**Figure 4:**
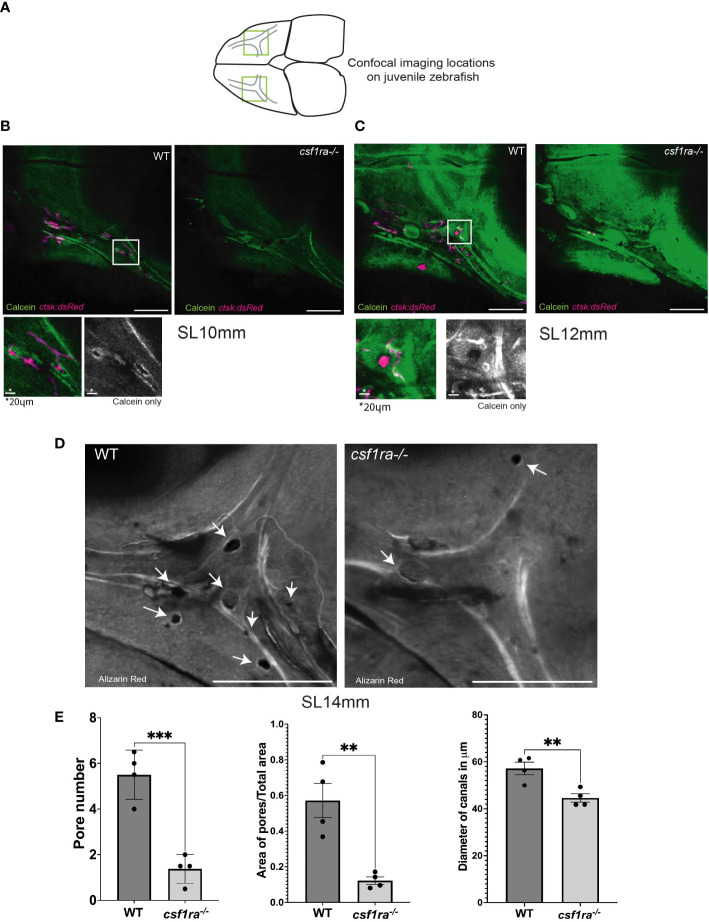
Imaging of supraorbital lateral line canals using calcein-stained *csf1ra* mutants with *ctsk:dsRed* and their wild-type siblings shows spatial associations between osteoclasts and foramina in the supraorbital lateral line canals. Individuals were size matched prior to imaging; all images are max projections of z-stacks and scale bars are 200μm unless otherwise indicated. **(A)** Highlighted areas on scheme represent locations of the confocal imaging conducted on the dorsal side of the skull in an area of high osteoclast activity as previously described in **Figure 2**. **(B)** SL10mm calcein-stained *ctsk:dsRed csf1ra^-/-^* mutants were live-imaged and wild type siblings show differences in osteoclast expression related to bone morphology. **(C)** SL12mm calcein-stained *ctsk:dsRed* live-imaged WT siblings and *csf1ra^-/-^* mutants show a continuing pattern from the earlier 10mm stage with activity around the pores **(D)** Bone from fixed alizarin red stained SL14mm individuals was imaged to capture a 3D representation, showing canals which lack pores in the mutant fish. **(E)** Quantification (WT n=4, *csf1ra^-/-^* n=4) of the number of pores (p=0.0006), followed by the area of the pores normalized using the total area (p=0.03). Measurements were taken from the canals on each frontal bone from the same individual. Since we cannot assume that the paired structures from each fish are independent these numbers were then averaged to generate one figure per individual prior to comparative analysis. Finally diameter of the canals at the juncture point was compared (p=0.008) with location of measurements taken marked in **(C)**, this generated 47 total measurements, 24 for the WT and 23 for the mutants (with one measurement missing due to irregularity of shape in mutant canal) the measurements from a single individual were averaged then statistical analysis conducted. For all charts lines indicate mean with SEM, Mann-Whitney U t-test (two-tailed) used for statistical analysis, 95% confidence interval. ** indicates p-value < 0.005, *** indicates p-value < 0.0005.

Our data indicate a role for osteoclasts in sculpting the hyomandibular foramen; in their absence, the canal is smaller early in ossification, and less complex later in development (**Figure 3**). To determine if osteoclasts are similarly required to shape the supraorbital lateral line canals, we compared *csf1ra* mutants and siblings at stages of highest osteoclast concentration. At SL10mm and SL12mm, osteoclasts are not distributed evenly throughout the canals (**Figures 4A, B**). Instead, they tend to associate with small pores in the bone in and around the canals. In the mutants at the same stages, the most striking difference is the almost total absence of pores. To compare the structural differences in detail, we conducted confocal imaging on dissected and Alizarin red stained frontal bones from mutants and siblings and generated z-stacks capturing the entirety of the area of interest. The *csf1ra^-/-^* mutants had significantly narrower anterior lateral line canals compared to stage–matched siblings (**Figure 4C**). More strikingly, mutants and siblings showed a highly statistically significant difference in the number and area of pores (**Figures 4D, E**).

## Discussion

We report here the first comprehensive description of osteoclast localization during vertebrate skull formation. Through serial live imaging, we described areas of high osteoclast concentration during development, and interrogated their functional roles in an osteoclast–deficient mutant.

Consistent with the motile nature of osteoclasts, their distribution is dynamic and varies within individual fish during development, and among fish at the same stage. We found the first osteoclasts marked by *ctsk:dsRed* line at around 5 dpf and this corresponds to previous research on osteoclast localization and bone formation during development in zebrafish (33, 55). Overall they are sparse, but tend to concentrate in several specific areas. To better visualize areas of concentration, we overlaid osteoclast positions from three separate fish, visualized from multiple angles and at several developmental stages. We observed areas of highest osteoclast concentration associated with cranial nerves and peripheral sensory cells, including the facial/auditory, lateral line nerves and the mandibular and supraorbital neuromasts (9).

One area with early clustering of osteoclasts was in and around the hyomandibular foramen, which allows passage of cranial nerves and thus connections with facial musculature, the inner ear and the neuromasts of the lateral line system (61–63). The foramen is already present in the hyomandibular cartilage, and is transversed by nerve fibers of facial nerve and aLL at 4 dpf (50). The final morphology of the hyomandibular foramen is shaped as development progresses in concert with ossification of the cartilage (6, 8). We find that the process of shaping is severely disrupted in the absence of osteoclasts in the *csf1ra* mutants. At the earliest stage of our imaging (SL6.8mm), the mutants have greatly reduced diameter of the foramen. By SL8mm, the foramen in the WT fish is more complex and curved, and while the diameter is increased in the mutants, the foramen retains a simple shape resembling the immature morphology.

Osteoclasts were also concentrated on the ventral side of the mandible and in the supraorbital canals, structures associated respectively with the mandibular and supraorbital neuromasts of the aLL (4). The formation of the supraorbital canals housing the neuromasts is quite different from the formation of the hyomandibular foramen. The neuromasts sit on the lateral edge of the frontal bone, but their pattern and distribution is established prior to its formation. Subsequently the underlying bone forms canals that envelop the neuromasts, with external openings to allow sensing of water pressure (36, 50, 64–66). Pores also form in the bone below the neuromasts and allow passage of nerve fibers (51). We find that both processes are disrupted in the absence of osteoclasts. The diameter of the canal is reduced during its formation, and more strikingly, the pores in the bone are largely absent. Our data highlights important parallels with the formation of the bony canals and the presence of osteoclasts around the posterior lateral line neuromasts (67). Positioning of neuromasts determine bone shape (59) and disruptions in neuromasts cause changes in the surrounding bone, implying a functional consequence (48, 50, 67). While we focused on two sites in the craniofacial skeleton, it is likely that the sculpting of bone associated with nerves is broadly disrupted in the absence of osteoclasts.

Prior work in many experimental systems has demonstrated the important instructive role of nerves in skeletal morphogenesis (37–39). Our results affirm the connection between nerves and bone, specifically during craniofacial development. We also demonstrate the critical role of osteoclasts in that connection. We speculate that possible signaling pathways involved could be CXCL12-CXCR4/CXCR7 (68–70) and/or Wnt-β-catenin (71, 72). Consistent with a broad role for osteoclasts in creating the scaffold for cranial nerves, patients with juvenile osteopetrosis frequently suffer neurologic sequelae associated with compression of the optic, facial, supraorbital and auditory nerves (27, 73–75). The overall architecture of the cranial nerves and their foramina are conserved between mammals and zebrafish (34, 76), which provides an accessible model to study these processes. Previous analysis of zebrafish *csf1ra* mutants has shown a general increase in bone mineral density, with an additive increase in fish also lacking the paralogue *csf1rb* (52), making these mutants a valuable model of osteoclast poor osteopetrosis. The presence of low amounts of *ctsk:dsRed* signal and smaller foramina in the mutants observed in our study (**Figure 3C**) could be attributed to the *csfr1b* paralogue. Homozygous *csf1ra* mutants are viable as adults, making feasible future examination of the hyomandibular and other foramina, and possible sequelae on nerve function and behavior. These detailed studies would expand the utility of the zebrafish model and provide insights into disrupted formation of cranial nerve foramina associated with osteopetrosis and other human diseases.

## Data availability statement

The datasets presented in this study can be found in online repositories. The names of the repository/repositories and accession number(s) can be found below: The daily live imaging and osteoclast overlay datasets generated for this study are publicly accessible on the FaceBase site, at https://www.facebase.org, FB00001169 and FB00001240.

## Ethics statement

The animal study was reviewed and approved by Boston University IACUC.

## Author contributions

SF was responsible for project supervision. KM was responsible for the experiments conducted. KM and AC were responsible for data analysis. JC-L and MH contributed the *cstk:dsRed* and *csf1ra^-/-^* lines and assisted in experimental design. KM, AC, MH, and SF contributed to the writing and editing of the manuscript. All authors contributed to the article and approved the submitted version.

## Funding

The work was supported by grants U01DE024434 to MH and SF, and R01DE022955 and R21DE021196 to SF from NIH/NIDCR, and T32GM008541 to KM.

## Acknowledgments

The authors would like to thank the staff, especially Derek Walsh, of the aquatics facility at Boston University for their care of our fish stocks. We would also like to thank Dr. Jacqueline F. Webb for helpful discussions.

## Conflict of interest

The authors declare that the research was conducted in the absence of any commercial or financial relationships that could be construed as a potential conflict of interest.

## Publisher’s note

All claims expressed in this article are solely those of the authors and do not necessarily represent those of their affiliated organizations, or those of the publisher, the editors and the reviewers. Any product that may be evaluated in this article, or claim that may be made by its manufacturer, is not guaranteed or endorsed by the publisher.

## References

[1] Abing W Rauchfuss A. Fetal development of the tympanic part of the facial canal. Arch Oto-rhino-laryngol (1987) 243:374–7. doi: 10.1007/BF00464645 3566620

[2] Edwards B Wang JM Iwanaga J Loukas M Tubbs RS. Cranial nerve foramina: Part II - a review of the anatomy and pathology of cranial nerve foramina of the posterior cranial fossa. Cureus (2018) 10:e2500. doi: 10.7759/cureus.2500 29928560 PMC6005399

[3] Twigg SR Wilkie AO. New insights into craniofacial malformations. Hum Mol Genet (2015) 24:R50–9. doi: 10.1093/hmg/ddv228 PMC4571997 26085576

[4] Teng CS Ting MC Farmer DT Brockop M Maxson RE Crump JG. Altered bone growth dynamics prefigure craniosynostosis in a zebrafish model of saethre-chotzen syndrome. Elife (2018) 7:e37024. doi: 10.7554/eLife.37024.041 30375332 PMC6207424

[5] Hamdan A-LH Nabulsi MM Farhat FT Haidar RK Fuleihan NS. When bone becomes marble: Head and neck manifestations of osteopetrosis. Paedia Child Health (2006) 11:37–40. doi: 10.1093/pch/11.1.37 PMC2435323 19030245

[6] Eames BF DeLaurier A Ullmann B Huycke TR Nichols JT Dowd J. FishFace: Interactive atlas of zebrafish craniofacial development at cellular resolution. BMC Dev Biol (2013) 13:23. doi: 10.1186/1471-213X-13-23 23714426 PMC3698193

[7] Mork L Crump G. Zebrafish craniofacial development: A window into early patterning. Curr Top Dev Biol (2015) 115:235–69. doi: 10.1016/bs.ctdb.2015.07.001 PMC4758817 26589928

[8] Cubbage CC Mabee PM. Development of the cranium and paired fins in the zebrafish danio rerio (Ostariophysi, cyprinidae). J Morphol (1996) 229:121–60. doi: 10.1002/(SICI)1097-4687(199608)229:2<121::AID-JMOR1>3.0.CO;2-4 29852585

[9] Kanther M Scalici A Rashid A Miao K Van Deventer E Fisher S. Initiation and early growth of the skull vault in zebrafish. Mech Dev (2019) 160:103578. doi: 10.1016/j.mod.2019.103578 31644945 PMC6988175

[10] Jacome-Galarza CE Percin GI Muller JT Mass E Lazarov T Eitler J. Developmental origin, functional maintenance and genetic rescue of osteoclasts. Nature (2019) 568:541–5. doi: 10.1038/s41586-019-1105-7 30971820 PMC6910203

[11] Kamakura T Nadol JBJ. Evidence of osteoclastic activity in the human temporal bone. Audiol Neurootol (2017) 22:218–25. doi: 10.1159/000481279 29224005

[12] Asagiri M Takayanagi H. The molecular understanding of osteoclast differentiation. Bone (2007) 40:251–64. doi: 10.1016/j.bone.2006.09.023 17098490

[13] Witten PE Huysseune A. A comparative view on mechanisms and functions of skeletal remodelling in teleost fish, with special emphasis on osteoclasts and their function. Biol Rev Camb Philos Soc (2009) 84:315–46. doi: 10.1111/j.1469-185X.2009.00077.x 19382934

[14] Kim JM Lin C Stavre Z Greenblatt MB Shim JH. Osteoblast-osteoclast communication and bone homeostasis. Cells (2020) 9:E2073. doi: 10.3390/cells9092073 32927921 PMC7564526

[15] Suda T Takahashi N Udagawa N Jimi E Gillespie MT Martin TJ. Modulation of osteoclast differentiation and function by the new members of the tumor necrosis factor receptor and ligand families. Endocr Rev (1999) 20:345–57. doi: 10.1210/edrv.20.3.0367 10368775

[16] Simonet WS Lacey DL Dunstan CR Kelley M Chang MS Lü̈thy R. Osteoprotegerin: a novel secreted protein involved in the regulation of bone density. Cell (1997) 89:309–19. doi: 10.1016/S0092-8674(00)80209-3 9108485

[17] Charles JF Aliprantis AO. Osteoclasts: more than ‘bone eaters’. Trends Mol Med (2014) 20:449–59. doi: 10.1016/j.molmed.2014.06.001 PMC4119859 25008556

[18] Helfrich MH. Osteoclast diseases. Microsc Res Tech (2003) 61:514–32. doi: 10.1002/jemt.10375 12879419

[19] Novack DV Teitelbaum SL. The osteoclast: friend or foe. Annu Rev Pathol (2008) 3:457–84. doi: 10.1146/annurev.pathmechdis.3.121806.151431 18039135

[20] To TT Witten PE Renn J Bhattacharya D Huysseune A Winkler C. Rankl-induced osteoclastogenesis leads to loss of mineralization in a medaka osteoporosis model. Development (2012) 139:141–50. doi: 10.1242/dev.071035 22096076

[21] To TT Witten PE Huysseune A Winkler C. An adult osteopetrosis model in medaka reveals the importance of osteoclast function for bone remodeling in teleost fish. Comp Biochem Physiol C Toxicol Pharmacol (2015) 178:68–75. doi: 10.1016/j.cbpc.2015.08.007 26334373

[22] Sobacchi C Frattini A Guerrini MM Abinun M Pangrazio A Susani L. Osteoclast-poor human osteopetrosis due to mutations in the gene encoding RANKL. Nat Genet (2007) 39:960–2. doi: 10.1038/ng2076 17632511

[23] Stattin EL Henning P Klar J McDermott E Stecksen-Blicks C Sandström. SNX10 gene mutation leading to osteopetrosis with dysfunctional osteoclasts. Sci Rep (2017) 7:3012. doi: 10.1038/s41598-017-02533-2 28592808 PMC5462793

[24] Van Wesenbeeck L Odgren PR Coxon FP Frattini A Moens P Perdu B. Involvement of PLEKHM1 in osteoclastic vesicular transport and osteopetrosis in incisors absent rats and humans. J Clin Invest (2007) 117:919–30. doi: 10.1172/JCI30328 PMC1838941 17404618

[25] Yetiser S. The dehiscent facial nerve canal. Int J Otolaryngol (2012) 2012:679708. doi: 10.1155/2012/679708 22518159 PMC3299328

[26] Nomiya S Kariya S Nomiya R Morita N Nishizaki K Paparella MM. Facial nerve canal dehiscence in chronic otitis media without cholesteatoma. Eur Arch Otorhinolaryngol (2014) 271:455–8. doi: 10.1007/s00405-013-2431-2 PMC3797195 23483192

[27] Chepla KJ Oh E Guyuron B. Clinical outcomes following supraorbital foraminotomy for treatment of frontal migraine headache. Plast Reconstr Surg (2012) 129:656e–62e. doi: 10.1097/PRS.0b013e3182450b64 PMC3315686 22456379

[28] Tomaszewska A Kwiatkowska B Jankauskas R. The localization of the supraorbital notch or foramen is crucial for headache and supraorbital neuralgia avoiding and treatment. Anatomical Rec Anat Rec (2012) 295:1494–503. doi: 10.1002/ar.22534 22807312

[29] Ealba EL Jheon AH Hall J Curantz C Butcher KD Schneider RA. Neural crest-mediated bone resorption is a determinant of species-specific jaw length. Dev Biol (2015) 408:151–63. doi: 10.1016/j.ydbio.2015.10.001 PMC4698309 26449912

[30] Edamoto M Kuroda Y Yoda M Kawaai K Matsuo K. Trans-pairing between osteoclasts and osteoblasts shapes the cranial base during development. Sci Rep (2019) 9:1956. doi: 10.1038/s41598-018-38471-w 30760811 PMC6374512

[31] Kimmel CB DeLaurier A Ullmann B Dowd J McFadden M. Modes of developmental outgrowth and shaping of a craniofacial bone in zebrafish. PloS One (2010) 5:e9475. doi: 10.1371/journal.pone.0009475 20221441 PMC2832765

[32] Witten PE Hansen A Hall BK. Features of mono- and multinucleated bone resorbing cells of the zebrafish danio rerio and their contribution to skeletal development, remodeling, and growth. J Morphol J Morphol (2001) 250:197–207. doi: 10.1002/jmor.1065 11746460

[33] Sharif F de Bakker MA Richardson MK. Osteoclast-like cells in early zebrafish embryos. Cell J (2014) 16:211–24. PMC4072079 24567948

[34] Higashijima S Hotta Y Okamoto H. Visualization of cranial motor neurons in live transgenic zebrafish expressing green fluorescent protein under the control of the islet-1 promoter/enhancer. J Neurosci (2000) 20:206–18. doi: 10.1523/JNEUROSCI.20-01-00206.2000 PMC6774115 10627598

[35] Thomas ED Cruz IA Hailey DW Raible DW. There and back again: development and regeneration of the zebrafish lateral line system. Wiley Interdiscip Rev Dev Biol (2015) 4:1–16. doi: 10.1002/wdev.160 25330982 PMC4268111

[36] Wada H Kawakami K. Size control during organogenesis: Development of the lateral line organs in zebrafish. Dev Growth Differ (2015) 57:169–78. doi: 10.1111/dgd.12196 25703577

[37] Abeynayake N Arthur A Gronthos S. Crosstalk between skeletal and neural tissues is critical for skeletal health. Bone (2021) 142:115645. doi: 10.1016/j.bone.2020.115645 32949783

[38] Elefteriou F. Impact of the autonomic nervous system on the skeleton. Physiol Rev (2018) 98:1083–112. doi: 10.1152/physrev.00014.2017 PMC6088147 29717928

[39] Tomlinson RE Christiansen BA Giannone AA Genetos DC. The role of nerves in skeletal development, adaptation, and aging. Front Endocrinol (Lausanne) (2020) 11:646. doi: 10.3389/fendo.2020.00646 33071963 PMC7538664

[40] Gerosa L Lombardi G. Bone-to-Brain: A round trip in the adaptation to mechanical stimuli. Front Physiol (2021) 12. doi: 10.3389/fphys.2021.623893 PMC8120436 33995117

[41] Wan Q-Q Qin W-P Ma Y-X Shen M-J Li J Zhang Z-B. Crosstalk between bone and nerves within bone. Advanced Sci Adv Sci (2021) 8:2003390. doi: 10.1002/advs.202003390 PMC8025013 33854888

[42] Mandl P Hayer S Karonitsch T. Nicotinic acetylcholine receptors modulate osteoclastogenesis. Arthritis Res Ther (2016) 18:63. doi: 10.1186/s13075-016-0961-x 26970742 PMC4789270

[43] Bajayo A Bar A Denes A Bachar M Kram V Attar-Namdar M. Skeletal parasympathetic innervation communicates central IL-1 signals regulating bone mass accrual. Proc Natl Acad Sci U S A (2012) 109:15455–60. doi: 10.1073/pnas.1206061109 PMC3458367 22949675

[44] Elefteriou F. Regulation of bone remodeling by the central and peripheral nervous system. Arch Biochem Biophys (2008) 473:231–6. doi: 10.1016/j.abb.2008.03.016 PMC2430105 18410742

[45] Schmidt R Strähle U Scholpp S. Neurogenesis in zebrafish - from embryo to adult. Neural Dev (2013) 8:3. doi: 10.1186/1749-8104-8-3 23433260 PMC3598338

[46] Tonelli F Bek JW Besio R De Clercq A Leoni L Salmon P. Zebrafish: A resourceful vertebrate model to investigate skeletal disorders. Front Endocrinol (Lausanne) (2020) 11:489. doi: 10.3389/fendo.2020.00489 32849280 PMC7416647

[47] Won SY Choi BO Chung KW Lee JE. Zebrafish is a central model to dissect the peripheral neuropathy. Genes Genomics (2019) 41:993–1000. doi: 10.1007/s13258-019-00838-2 31183681

[48] Chang CT Franz-Odendaal TA. Perturbing the developing skull: using laser ablation to investigate the robustness of the infraorbital bones in zebrafish (Danio rerio). BMC Dev Biol (2014) 14:44. doi: 10.1186/s12861-014-0044-7 25516292 PMC4282728

[49] Piotrowski T Baker CV. The development of lateral line placodes: taking a broader view. Dev Biol (2014) 389:68–81. doi: 10.1016/j.ydbio.2014.02.016 24582732

[50] Wada H Ghysen A Satou C Higashijima S Kawakami K Hamaguchi S. Dermal morphogenesis controls lateral line patterning during postembryonic development of teleost fish. Dev Biol (2010) 340:583–94. doi: 10.1016/j.ydbio.2010.02.017 20171200

[51] Webb JF Shirey JE. Postembryonic development of the cranial lateral line canals and neuromasts in zebrafish. Dev Dyn (2003) 228:370–85. doi: 10.1002/dvdy.10385 14579376

[52] Caetano-Lopes J Henke K Urso K Duryea J Charles JF Warman ML. Unique and non-redundant function of csf1r paralogues in regulation and evolution of post-embryonic development of the zebrafish. Development (2020) 147:dev181834. doi: 10.1242/dev.181834 31932352 PMC6983717

[53] Westerfield M. “General methods for Zebrafish care”. Eugene: Univ Oregon Press (2007).

[54] Chatani M Takano Y Kudo A. Osteoclasts in bone modeling, as revealed by *in vivo* imaging, are essential for organogenesis in fish. Dev Biol (2011) 360:96–109. doi: 10.1016/j.ydbio.2011.09.013 21963458

[55] Du SJ Frenkel V Kindschi G Zohar Y. Visualizing normal and defective bone development in zebrafish embryos using the fluorescent chromophore calcein. Dev Biol (2001) 238:239–46. doi: 10.1006/dbio.2001.0390 11784007

[56] Parichy DM Elizondo MR Mills MG Gordon TN Engeszer RE. Normal table of postembryonic zebrafish development: staging by externally visible anatomy of the living fish. Dev Dyn (2009) 238:2975–3015. doi: 10.1002/dvdy.22113 19891001 PMC3030279

[57] Walker MB Kimmel CB. A two-color acid-free cartilage and bone stain for zebrafish larvae. Biotech Histochem (2007) 82:23–8. doi: 10.1080/10520290701333558 17510811

[58] Kague E Roy P Asselin G Hu G Simonet J Stanley A. Osterix/Sp7 limits cranial bone initiation sites and is required for formation of sutures. Dev Biol (2016) 413:160–72. doi: 10.1016/j.ydbio.2016.03.011 PMC5469377 26992365

[59] Powers AK Boggs TE Gross JB. Canal neuromast position prefigures developmental patterning of the suborbital bone series in astyanax cave- and surface-dwelling fish. Dev Biol (2018) 441:252–61. doi: 10.1016/j.ydbio.2018.04.001 PMC6119090 29630866

[60] Herbomel P Thisse B Thisse C. Zebrafish early macrophages colonize cephalic mesenchyme and developing brain, retina, and epidermis through a m-CSF receptor-dependent invasive process. Dev Biol (2001) 238:274–88. doi: 10.1006/dbio.2001.0393 11784010

[61] Iwasaki M Yokoi H Suzuki T Kawakami K Wada H. Development of the anterior lateral line system through local tissue-tissue interactions in the zebrafish head. Dev Dyn (2020) 249:1440–54. doi: 10.1002/dvdy.225 32658373

[62] Crucke J Van de Kelft A Huysseune A. The innervation of the zebrafish pharyngeal jaws and teeth. J Anat (2015) 227:62–71. doi: 10.1111/joa.12321 26018453 PMC4475359

[63] Sheets L Holmgren M Kindt KS. How zebrafish can drive the future of genetic-based hearing and balance research. J Assoc Res Otolaryngol (2021) 22:215–35. doi: 10.1007/s10162-021-00798-z PMC8110678 33909162

[64] Alexandre D Ghysen A. Somatotopy of the lateral line projection in larval zebrafish. Proc Natl Acad Sci USA (1999) 96:7558–62. doi: 10.1073/pnas.96.13.7558 PMC22125 10377454

[65] Ghysen A Dambly-Chaudière C. The lateral line microcosmos. Genes Dev (2007) 21:2118–30. doi: 10.1101/gad.1568407 17785522

[66] Klein A Bleckmann H. Function of lateral line canal morphology. Integr Zool (2015) 10:111–21. doi: 10.1111/1749-4877.12101 24920149

[67] Wada H Iwasaki M Kawakami K. Development of the lateral line canal system through a bone remodeling process in zebrafish. Dev Biol (2014) 392:1–14. doi: 10.1016/j.ydbio.2014.05.004 24836859

[68] Dambly-Chaudière C Cubedo N Ghysen A. Control of cell migration in the development of the posterior lateral line: antagonistic interactions between the chemokine receptors CXCR4 and CXCR7/RDC1. BMC Dev Biol (2007) 7:23. doi: 10.1186/1471-213X-7-23 17394634 PMC1847803

[69] David NB Sapède D Saint-Etienne L Thisse C Thisse B Dambly-Chaudière C. Molecular basis of cell migration in the fish lateral line: Role of the chemokine receptor CXCR4 and of its ligand, SDF1. Proc Natl Acad Sci (2002) 99:16297–302. doi: 10.1073/pnas.252339399 PMC138605 12444253

[70] Shahnazari M Chu V Wronski TJ Nissenson RA Halloran BP. CXCL12/CXCR4 signaling in the osteoblast regulates the mesenchymal stem cell and osteoclast lineage populations. FASEB J (2013) 27:3505–13. doi: 10.1096/fj.12-225763 23704087

[71] Kobayashi Y Uehara S Koide M Takahashi N. The regulation of osteoclast differentiation by wnt signals. Bonekey Rep (2015) 4:713. doi: 10.1038/bonekey.2015.82 26157576 PMC4495780

[72] Jacques BE Montgomery WH Uribe PM Yatteau A Asuncion JD Resendiz G. The role of wnt/β-catenin signaling in proliferation and regeneration of the developing basilar papilla and lateral line. Dev Neurobiol (2014) 74:438–56. doi: 10.1002/dneu.22134 24115534

[73] Benecke JE. Facial nerve dysfunction in osteopetrosis. Laryngoscope (1993) 103:494–7. doi: 10.1288/00005537-199305000-00002 8483364

[74] McGonnell IM Akbareian SE. Like a hole in the head: Development, evolutionary implications and diseases of the cranial foramina. Semin Cell Dev Biol (2019) 91:23–30. doi: 10.1016/j.semcdb.2018.08.011 30385045

[75] Steward CG. Neurological aspects of osteopetrosis. Neuropathol Appl Neurobiol (2003) 29:87–97. doi: 10.1046/j.1365-2990.2003.00474.x 12662317

[76] Mueller T Vernier P Wullimann MF. The adult central nervous cholinergic system of a neurogenetic model animal, the zebrafish danio rerio. Brain Res (2004) 1011:156–69. doi: 10.1016/j.brainres.2004.02.073 15157802

