# Supplementary material for: Osteoclast activity sculpts craniofacial form to permit sensorineural patterning in the zebrafish skull
Source: Front Endocrinol (Lausanne). 2022 Nov 1;13:969481. doi: 10.3389/fendo.2022.969481 (PMC9664155; doi:10.3389/fendo.2022.969481)
Supplement: Supplementary file 1 [file DataSheet_1.docx]

## Supplementary Figures


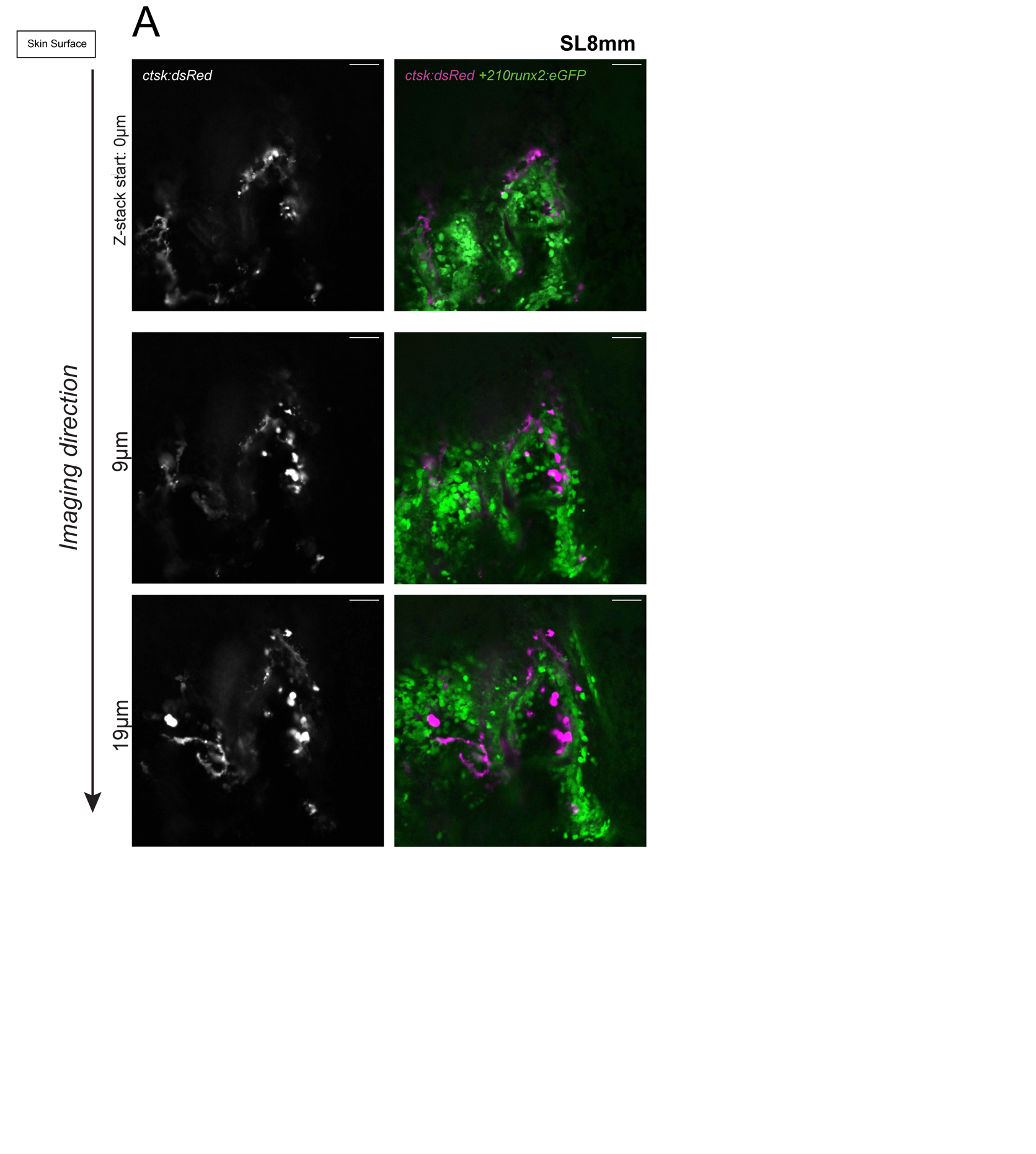


**Supplementary Figure 1.** 8mm *ctsk:dsRed* and *+210runx2:eGFP* transgenic fish, the latter a marker for osteoblasts, were euthanized by rapid cooling, followed by fixation in cold 4% PFA in PBS for 20 minutes. Heads were removed and mounted into glass bottom dishes with 2% molecular-grade agarose. Samples were imaged on a Zeiss LSM 710-Live Duo Scan microscope in the Boston University School of Medicine Cellular Imaging Core. All scale bars represent 50μm A) Representative images selected from the Z-stack of a single imaged SL8mm individual of the hyomandibular foramen moving down into the tissue starting from skin surface. Close spatial associations between osteoclasts and osteoblasts can be observed especially at the middle 9μm depth. At the 19μm depth a concentration of osteoclast cells can be seen within the open spaces of the canal as delineated in this imaging series by the lack of osteoblasts.
